# Identification and genetic characterization of a novel species of *Choleoeimeria* Schneider, 1875 from a captive‐bred bilby (Thylacomyidae; *Macrotis lagotis*) (Reid, 1837) in Western Australia

**DOI:** 10.1002/ece3.10933

**Published:** 2024-02-20

**Authors:** Belinda Brice, Huimin Gao, Bruno P. Berto, Gwyneth Thomas, Aileen Elloit, Alireza Zahedi

**Affiliations:** ^1^ Kanyana Wildlife Rehabilitation Centre Lesmurdie Western Australia Australia; ^2^ Institute of Cash Crops Hebei Academy of Agriculture and Forestry Sciences Shijiazhuang China; ^3^ Departamento de Biologia Animal, Instituto de Ciências Biológicas e da Saúde Universidade Federal Rural do Rio de Janeiro Seropédica Rio de Janeiro Brazil; ^4^ College of Science, Health, Engineering and Education Murdoch University Murdoch Western Australia Australia; ^5^ The Centre of Biosecurity and One Health, Harry Butler Institute Murdoch University Perth Western Australia Australia

**Keywords:** 18S rRNA, bilby, *Choleoeimeria*, coccidia, COI, *Macrotis lagotis*

## Abstract

A novel *Eimeria* sp. from a captive‐bred bilby (*Macrotis lagotis* Reid, 1837) has been identified in Western Australia. The bilby was bred at the Kanyana Wildlife Rehabilitation Centre, Perth, as part of the National Bilby Recovery Plan. Oocysts (*n* = 31) irregular blunt ellipsoidal, 17–18 × 11–12 (17.2 × 11.3); length/width (L/W) ratio 1.4–1.5 (1.5). Wall bi‐layered, 0.8–1.0 (0.9) thick, outer layer smooth, c.2/3 of total thickness. Micropyle barely discernible. Oocyst residuum is absent, but 2–3 small polar granules are present. Sporocysts (*n* = 31) ovoidal, 7–8 × 5–6 (7.8 × 5.7); L/W ratio 1.3–1.4 (1.4). Stieda, sub‐Stieda and para‐Stieda bodies absent or indiscernible; sporocyst residuum present, usually as an irregular body consisting of numerous granules that appear to be membrane‐bound or sometimes diffuse among sporozoites. Sporozoites vermiform with a robust refractile body. Further molecular characterization was conducted on the sporulated oocysts. At the 18S locus, it sat in a large clade of the phylogenetic tree with two isolates of *Eimeria angustus* from quendas (*Isoodon obesulus* Shaw, 1797) and the *Choleoeimeria* spp. It shared the highest identity with *E. angustus* (KU248093) at 98.84%; at the COI gene locus, it was unique and most closely related to *Choleoeimeria taggarti*, which is hosted by another species of marsupial, the yellow‐footed antechinus (*Antechinus flavipes flavipes*), with 90.58% genetic similarity. Based on morphological and molecular data, this isolate is a new species and named as *Choleoeimeria yangi* n. sp.

## Graphical abstract

A new species of Choleoeimeria from a captive‐bred bilby (*Macrotis lagotis* Reid, 1837) has been identified and described using morphological and molecular information.

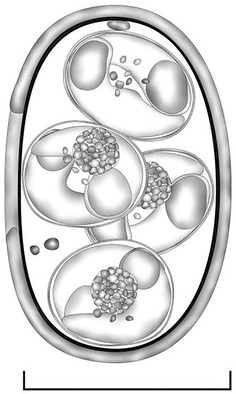

## 1 INTRODUCTION

The greater bilby (*Macrotis lagotis* Reid, 1837) is a ground‐dwelling Australian marsupial. This nocturnal animal belongs to the subclass Marsupialia, the order Peramelemorphia, and is a member of the Thylacomyidae family. Originally, there were two bilby species, namely the greater bilby and the lesser bilby (*Macrotis leucura*) (Thomas, 1887) but the latter bilby species became extinct between the 1930s and 1950s (Pavey, 2006). The greater bilby or bilby, as it is now known, has many common names, including Dalgyte, Rabbit‐eared bandicoot, Ninu or Walpajirra (Butcher, 2003).

Bilbies were once commonly found on around 70% of the Australian continent but are now limited to about 20% (Southgate, 1990). They are now only found in small, fragmented, isolated pockets of spinifex grasslands and mulga shrublands in the Tanami Desert in the Northern Territory, the Great Sandy Desert in the Kimberley, Gibson Desert, the Pilbara of Western Australia and in the Mitchell grasslands of southwest Queensland (Southgate, 1990). Habitat loss and competition for their habitat with introduced species such as sheep, cattle and European rabbits (*Oryctolagus caniculus* Linnaeus, 1758), as well as demand for agricultural land, has led to a huge reduction in both their home range and their numbers over the last century (Pavey, 2006). Other introduced species like feral cats (*Felis silvestris* Schreber, 1777) and red foxes (*Vulpes vulpes* Linnaeus, 1758) also prey on native wildlife, including bilbies. Changes in fire patterns have also contributed to their decline (Pavey, 2006).

The bilby is listed as Vulnerable on the IUCN red list (Burbidge & Woinarski, 2016) and is protected throughout Australia. A National Bilby Recovery Plan (Pavey, 2006) has been implemented which includes breeding the bilby in captivity with the aim of re‐establishing bilbies in enclosed predator proof areas. The Kanyana Wildlife Rehabilitation Centre (KWRC) in Perth, Western Australia, is one of the captive breeding centres tasked with the important job of breeding these animals in captivity.

Coccidia are a diverse, complex group of protozoan parasites which are known to infect a wide range of vertebrate hosts, including marsupials (Tenter et al., 2002). To date, numerous *Eimeria* spp. have been detected in a wide range of marsupial hosts encompassing several marsupial families including the Dasyuridae, Vombatidae, Macropodidae, Potoroidae, Phalangeridae and Peramelidae; however, no *Eimeria* spp. have been reported from the Thylacomyidae family. *Choleoeimeria*, as a new coccidian genus, was first proposed by Paperna and Landsberg (1989) to include tetrasporocystic, dizoic and *Eimeria*—like coccidians infecting reptilian gall bladder epithelium (El‐Toukhy et al., 2014; Yang et al., 2015).

More than 50 *Eimeria* spp. have been described from members of the Macropodidae family. These include *Eimeria* spp. in the western grey kangaroo (*Macropus fuliginosus* Desmarest, 1817), eastern grey kangaroo (*Macropus giganteus* Shaw, 1790), black‐striped wallaby (*Macropus dorsalis* Gray, 1837), tammar wallaby (*Macropus eugenii* Desmarest, 1817), euro (*Macropus robustus* Gould, 1841), western brush wallaby (*Macropus irma* Jourdan, 1837), whip‐tailed wallaby (*Macropus parryi* Bennet, 1835), parma wallaby (*Macropus parma* Waterhouse, 1845), the agile wallaby (*Macropus agilis* Gould, 1842) (Barker et al., 1989), rock wallaby (*Petrogale* spp. Gray, 1827) (Barker et al., 1988b; O'Callaghan et al., 1998), red‐necked wallaby (*Macropus rufogriseus* Desmarest, 1817) (Ekawasti et al., 2020) and in the quokka (*Setonix brachyurus* Quoy and Gaimard, 1830) (Austen et al., 2014; Barker et al., 1988a).

*Eimeria kanyana* was described from the western barred bandicoot (*Perameles bougainville* Quoy and Gaimard, 1824) by Bennet et al. (2006) and *Eimeria angustus* and *Eimeria quenda* from the southern brown bandicoot (*Isoodon obesulus* Shaw, 1797) by Hillman et al. (2016). Both these small marsupials are members of the Peramelidae family. *Eimeria* spp. including *Eimeria potoroi* (Barker et al., 1988b) have been morphologically described from potoroid marsupials including *Eimeria burdi* from the boodie (*Bettongia lesueur* Quoy and Gaimard, 1824) (Hulst et al., 2016). *Eimeria taggarti* was isolated from the prostate of an antechinus (*Antechinus flavipes* Macleay, 1841), a member of the Dasyuridae family, by Amery‐Gale et al. (2018).

Within their varying host range, *Eimeria* spp. exhibit variation in shape and size of their oocysts, making it difficult to identify new species using traditional methods alone. It is essential to use both traditional methodologies as well as modern molecular techniques to accurately delimit species (Tenter et al., 2002). Despite *Eimeria* spp. being detected in a wide range of marsupials (Barker et al., 1988a, 1988b, 1989), limited genetic analyses of these coccidian parasites are available. Some of the *Eimeria* spp. that have been genetically characterized include *Eimeria trichosuri*, from brushtail possums (*Trichosurus vulpecula* Kerr, 1792), first reported by O'Callaghan and O'Donoghue (2001) and genetically characterized by Power et al. (2009). *Eimeria quokka* and *Eimeria setonicis* were first characterized by Barker et al. (1988b) and later were further characterized both morphologically and molecularly by Austen et al. (2014). *Eimeria woyliei* has been morphologically and genetically characterized from the critically endangered woylie (*Bettongia penicillata*, Gray, 1837) by Northover et al. (2019). In addition, Yang et al. (2012) attempted to both morphologically and genetically characterize *Eimeria* spp. from euros, red kangaroos (*Osphranter rufus* Desmarest, 1822) and western grey kangaroos. However, only *Eimeria wilcanniensis* from a red kangaroo was successfully identified. The oocysts from most of the faecal samples could not be detected under the microscope. Therefore, only the 18S rRNA sequences were amplified from those samples.

So far, only one *Choleoeimeria* sp. was reported from marsupials (*Choleoeimeria taggarti*) (Kruth et al., 2020).

Previously reported protozoan parasites reported to infect bilbies include *Cryptosporidium muris*, identified in a captive breeding colony of bilbies in 2003 (Warren et al., 2003). *Sarcocystis* spp. have also been recorded and were associated with necrotic foci in the pancreas, adrenal gland and liver. *Klossiella* spp. were detected as an incidental finding as well as *Toxoplasma gondii* (Vogelnest & Woods, 2008).

It is important that we understand the role played by parasites and diseases during the release or translocation process of captive‐bred animals, including bilbies, as they may play a role in the outcome of such projects. In the present study, we characterized *Choleoeimeria yangi* n. sp., both morphologically and genetically, from a captive‐bred Australian bilby. This is the first *Eimeria* species to be reported and both morphologically and genetically characterized from the Thylacomyidae family of marsupials.

## 2 MATERIALS AND METHODS

### 2.1 Sample collection and examination

Faecal samples were collected for routine gastrointestinal parasite screening from captive bilbies that were part of the KWRC bilby breeding programme. Microscopy at the KWRC is carried out by experienced microscopy volunteers. Samples included those from a juvenile, captive‐bred bilby. This female bilby, one of twins, was first seen in its mothers' pouch in June 2020. A few unsporulated oval oocysts were seen in a saline wet mount. A faecal float, using a sodium nitrate (NaNO_3_) faecal flotation solution (Apex Laboratories, NSW, Australia), was also performed. A portion of the faecal sample containing coccidian oocysts was placed in 2% potassium dichromate (K_2_O_2_O_7_) and taken to Murdoch University for further study. Between January 2016 and March 2021, 126 faecal samples from 30 captive bilbies (18 male and 12 female) at the KWRC were screened for intestinal parasites.

### 2.2 Morphological analysis

The sample preparation for the morphological study was as described by Yang et al. (2023). Briefly, a portion of the emulsified faecal sample solution was poured into the base of a Petri dish, to a depth of less than a cm, and stored in a cupboard in the dark, at room temperature (20–22°C). Sporulated oocysts were observed using an Olympus DP71 digital microimaging camera. Images were taken using Nomarski contrast with a 100× oil immersion objective.

### 2.3 DNA isolation

Total DNA was extracted from 200 mg of each faecal sample using a DNeasy PowerSoil Pro Kit (Clayton, VIC 3168, Australia) according to the manufacturer's instruction, with a slight modification. Prior to the start of the DNA extraction, the samples were subjected to four cycles of freezing/thawing using liquid nitrogen and boiling water to physically break down the oocyst wall, which is extremely resistant to chemical and mechanical disruption.

### 2.4 PCR amplification and sequencing

A nested PCR for the 18S rRNA amplification was conducted according to the protocols described by Yang, Brice, Elloit, and Ryan (2016) and Yang, Brice, and Ryan (2016). Briefly, the nested PCR with the primers EiGTF1 5′‐TTC ACA GGA CCC TCC GAT C and EIGTR1 5′‐AAC CAT GGT AAT TCT ATG G was used for the external amplification of the 18S rRNA sequence. The expected PCR product was ~1510 bp. The primers EiGTF2 5′‐TTA CGC CTA CTA GGC ATT CC and EiGTR2 5′‐TGA CCT ATC AGC TTT CGA CG were used for the internal reaction. The PCR reaction contained 10 μL of 2× GoTaq PCR master mix, 10 pM of each primer, (Promega, Sydney, NSW), 1 μL of DNA (~50 ng) for the external reaction or 1 μL of external PCR product for the internal reaction, and 7 μL of H_2_O. PCR cycling conditions both for the external and internal reactions were 1 cycle of 94°C for 3 min, followed by 40 cycles of 94°C for 30 s, 58°C for 30 s and 72°C for 2 min and a final extension of 72°C for 5 min. A partial mitochondrial cytochrome oxidase gene (COI) sequence (637 bp) was amplified using a nested PCR with the protocol described by Yang, Brice, and Ryan (2016). Briefly, the cytochrome oxidase gene (COI) gene sequence (723 bp) was amplified using the external primers COIF1 (5′ GGTTCAGGTGTTGGTTGGAC 3′) and COIF2 (5′ TAAGTACATCCCTAATGTC 3′) and the internal primers COIR1 (5′ CCAAGAGATAATACRAARTGGAA 3′) and COIR2 (5′ ATAGTATGTATCATGTARWGCAA 3′). The PCR reactions and conditions were the same as per the 18S PCR carried out for the bilby samples except for a shorter 72°C final 1‐min PCR extension step.

The PCR products were purified using a filter tip method as previously described by Yang et al., 2013 on an agarose gel and directly sequenced in both directions using each PCR primer with the ABI PrismTM Terminator V3.1 Cycle Sequencing kit (Applied Biosystems, Foster City, California). Primers were used with a final concentration of 3.3 mM in a final volume of 10 μL. The cycling conditions consisted of 25 cycles at 94°C for 30 s, 55°C for 5 s and 60°C for 4 min. The products of the sequencing reactions were cleaned up using a standard ethanol sodium acetate precipitation protocol by adding 1/10 volume of 3 M sodium acetate (pH 5.2) and 2× volume of 100% ethanol. They were mixed well by inversion and incubated at −20°C for 2 h. The Eppendorf tubes were centrifuged at 14,000 rpm at 4°C for 15 min to collect the pellets (indistinct), which were then washed with 100 μL 75% ethanol, followed by centrifugation for 5 min at room temperature. The Eppendorf tubes were dried in a SpeedVac for 30 min and the sequencing products were ready for sequencing.

### 2.5 Phylogenetic analysis

The raw sequencing data were edited and aligned using Finch TV® v1.4.0. (http://www.geospiza.com/Products/finchtv.shtml). Phylogenetic trees were constructed for *Eimeria* spp. at the 18S and COI loci with additional isolates from GenBank. Maximum likelihood (ML) analysis was conducted using MEGA‐X (Molecular Evolutionary Genetics Analysis) software (with the method of Tamura‐Nei based on the most appropriate model selection [TN92 + G + I for 18S rRNA and GRT + G + I for COI gene, respectively] using ModelTest in MEGA‐X). Bootstrap analyses were conducted using 1000 replicates to assess the reliability of inferred tree topologies.

### 2.6 Line drawing

Line drawings were edited using two software applications from CorelDRAW® (Corel Draw Graphics Suite, Version 2020, Corel Corporation, Canada), i.e., Corel DRAW and Corel PHOTO‐PAINT (Yang et al., 2021).

## 3 RESULTS

### 3.1 Morphologic description of *C. yangi* n. sp.

#### 3.1.1 Species description

**FIGURE 1:**
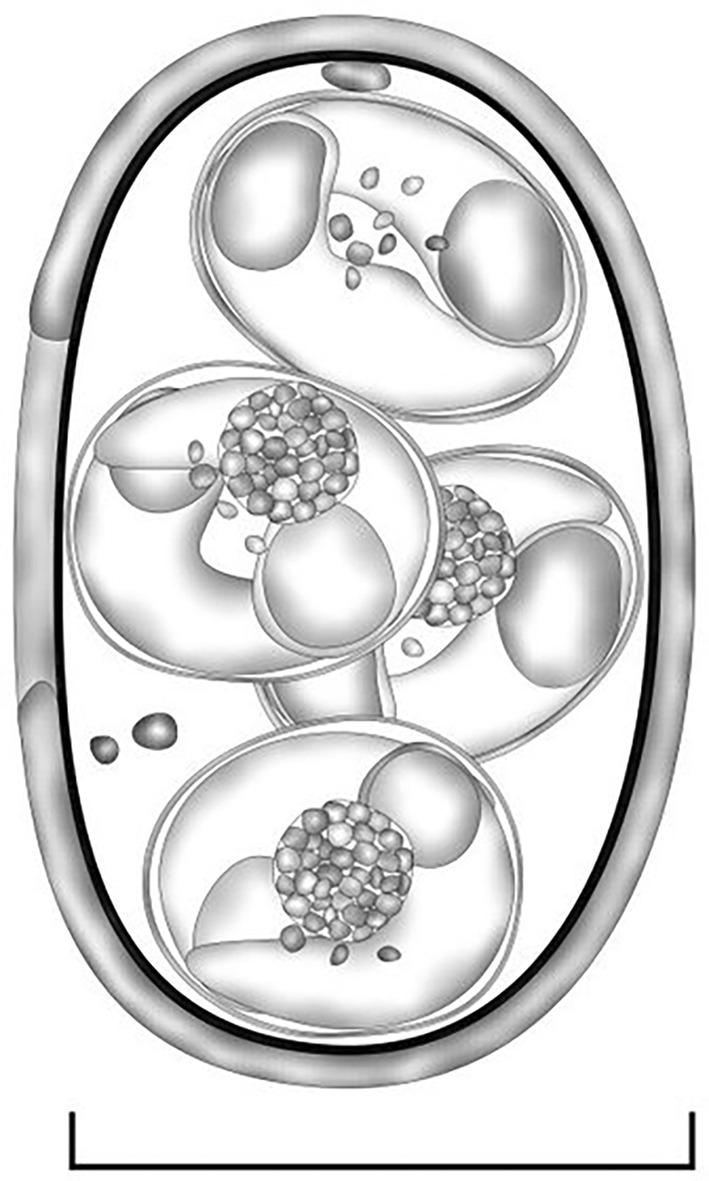
Composite line drawing of *Choleoeimeria yangi* n. sp. sporulated oocyst. Scale bar = 20 μm.

**FIGURE 2:**
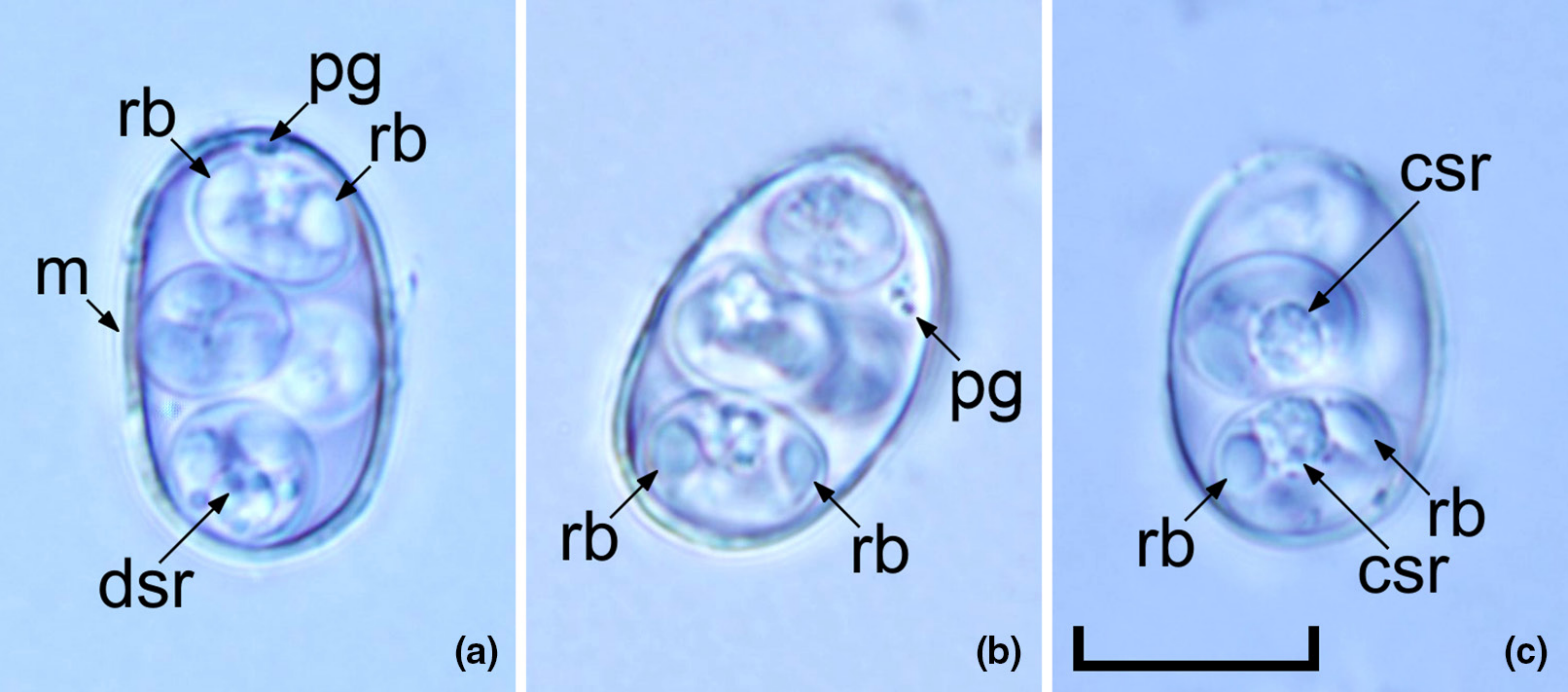
Photomicrographs of sporulated oocysts of *Choleoeimeria yangi* n. Sp. from the bilby *Macrotis lagotis*. Note the micropyle (m), polar granule (pg), refractile body (rb) and diffuse (dsr) or compact (csr) sporocyst residuum. Scale bar: 10 μm.

Oocysts (*n* = 31) irregular blunt ellipsoidal, 17–18 × 11–12 (17.2 × 11.3); length/width (L/W) ratio 1.4–1.5 (1.5). Wall bi‐layered, 0.8–1.0 (0.9) thick, outer layer smooth, c.2/3 of total thickness. Micropyle barely discernible. Oocyst residuum absent, but 2–3 small polar granules are present. Sporocysts (*n* = 31) ovoidal, 7–8 × 5–6 (7.8 × 5.7); L/W ratio 1.3–1.4 (1.4). Stieda, sub‐Stieda and para‐Stieda bodies absent or indiscernible; sporocyst residuum present, usually as an irregular body consisting of numerous granules that appear to be membrane‐bound, or sometimes diffuse among sporozoites. Sporozoites vermiform, with a robust refractile body (Figures 1 and 2).

Type hosts: Greater bilby (*Macrotis lagotis* Reid, 1837).

Type locality: Perth (−31.953512S, 115.857048E), Western Australia.

Prevalence: 3.3% (1 out of 30 captive bilbies).

Other hosts: Unknown.

Prepatent period: Unknown.

Patent period: Unknown.

Site of infection: Unknown.

Sporulation time: 48–72 h.

Material deposited: The hapantotype of the oocysts in 10% formalin and oocyst photosyntypes were deposited in the Western Australian Museum under the reference number WAM Z68802.

DNA sequences obtained from this study have been submitted to GenBank and the accession numbers are OR543995 and ON894305, respectively.

*Etymology*: This species is named *Choleoeimeria yangi* n. sp. after Dr Rongchang Yang, a molecular parasitologist from Murdoch University, Australia. Rongchang Yang, as one of the pioneers of coccidian molecular taxonomy worldwide, has established a standard protocol to characterize coccidian parasites in Australian wildlife both morphologically and molecularly. To date, over 30 new coccidian parasites have been named and described by his team.

### 3.2 Genetic characterization of *Choleoeimeria yangi* n. sp.

**FIGURE 3:**
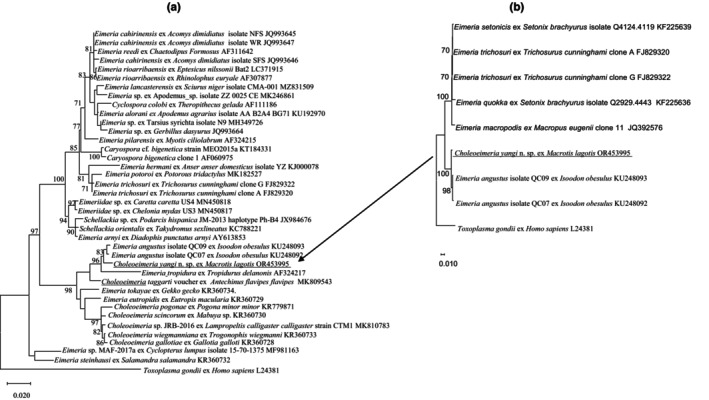
(a) Evolutionary relationships of *Choleoeimeria yangi* n. sp. inferred by distance analysis of 18S rRNA sequences. Percentage support (>70%) from 1000 pseudoreplicates from ML analysis based on a 1211 bp. (b) Phylogenetic relationships of *C. yangi* n. sp. and three additional *Eimeria* spp. from the Australian marsupia (*E. setonicis*, *E. quokka* and *E. quokka*) (1159 bp only).

**FIGURE 4:**
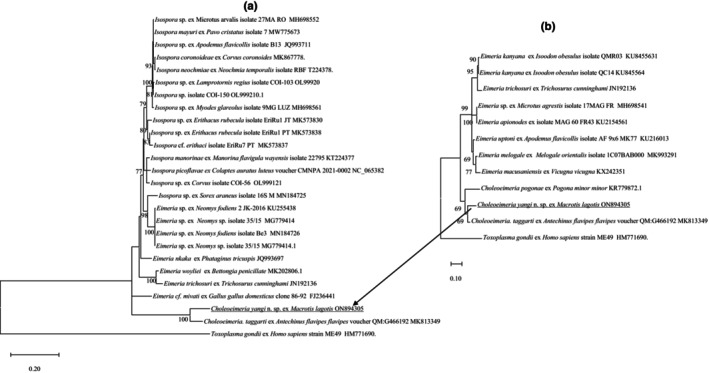
(a) Evolutionary relationships of *Choleoeimeria yangi* n. sp. inferred by distance analysis of COI sequences. Percentage support (>70%) from 1000 pseudoreplicates from ML analysis based on a 634 bp. (b) Phylogenetic relationships of *C. yangi* n. sp., *Choleoeimeria taggarti*, *Choleoeimeria pogonae and* eight additional *Eimeria* spp. from the Australian marsupia (178 bp only).

The phylogeny of *C. yangi* n. sp. was conducted using ML analyses at two gene loci (18S rRNA and COI). An alignment was generated for the 18S rRNA locus (1211 bp, Figure 3a) and an alignment for the COI locus (637 bp, Figure 4a). All alignments contained the sequence from *C. yangi* n. sp. as well as the representative reference sequences downloaded from GenBank including an outgroup (*Toxoplasma gondii*). In addition, due to some short versions of the 18S rRNA and COI sequences of *Choleoeimeria* spp. and *Eimeria* spp. from marsupials available in GenBank, a sub‐tree for 18S rRNA and COI are also given to show the relationship between *C. yangi* and other coccidian species. Similar phylogenetic relationships were observed between both loci, for example, both the 18S rRNA and COI of *C. yangi* n. sp. were most close to the *Choleoeimeria* spp. and *Eimeria* spp. identified from other marsupials (Figures 3a and 4a).

### 3.3 18S rRNA locus

*Choleoeimeria yangi* n. sp. shared the highest genetic similarity with *E. angustus* (KU248093) at 98.84% isolated from the southern brown bandicoot (quenda) (*Isoodon obesulus*) (Hillman et al., 2016) and followed by 97.6% genetic similarity with *Choleoeimeria taggarti* (MK809543), which was identified from the yellow‐footed antechinus (*Antechinus flavipes*) in Australia (Kruth et al., 2020).

### 3.4 COI gene locus

Pairwise comparison of the COI locus (637 bp) revealed that *C. yangi* n. sp. shared the highest genetic similarity of 90.6% with *C. taggarti* (MK813349), followed by various *Isospora* spp. with a genetic similarity between 80.4% and 79.7%. The *Choleoeimeria* spp., *Eimeria* spp. and *Isospora* spp. grouped into three separate clades except an unnamed *Isospora* sp. isolated from a common shrew (*Sorex araneus* Linnaeus, 1758) (MN184725), which sat in the *Eimeria* clade (Figure 4a). A *Choleoeimeria sequence* from *Choleoeimeria pogonae*, which was isolated from a Western bearded dragon (*Pogona minor minor*) (Yang et al., 2015), was added in the sub‐tree analysis (Figure 4b). It revealed that *C. yangi* n. sp. sat with the other two *Choleoeimeria* sequences in a separate clade.

## 4 DISCUSSION

After screening many faecal samples from captive bilbies at the KWRC, persistence finally paid off and a novel *Choleoeimeria* sp. was identified from the bilby. Faecal testing of the bilbies at the KWRC is routinely done every 6 weeks by experienced volunteer microscopists. No harmful parasites or bacteria have been found, except in the *C. muris* episode according to KWRC founder J. Butcher (2005). Nematode eggs and/or larvae and *Trichomonas* spp. have been reported during routine faecal screening at KWRC, but this is a very rare occurrence (Brice, personal communication, 2023).

In this study, the incidence of coccidia in captive bilbies at KWRC was 3.3%. Only one bilby over a 10‐year period was found to be infected with coccidia. The female juvenile bilby in this study was one of a pair of twins. It appeared healthy and exhibited no signs of a coccidian infection. Faecal samples from the sibling and the parents were repeatedly negative for coccidian oocysts (saline wet mount and faecal float). It is possible that the oocysts in the bilby in this study were prevalent at very low numbers in a parent/parents and that these were missed by faecal float. Both parents were healthy and showed no signs of coccidian infection. A personal communication by G. Wilson to K. A. Johnson (1989, in *Fauna of Australia*, chapter 25), reported the results of a study on 271 faecal samples collected from captive bilbies. 15/271 were positive for nematode eggs or larvae whilst no protozoa or coccidian oocysts were observed in approximately 200 wet preparations studied. These results suggest a low gastrointestinal parasite rate in bilbies. The incidence of coccidia in wild bilby populations is not known and further research on the infectious diseases affecting threatened bilbies would be most valuable.

To our knowledge, this is the first *Choleoeimeria* sp. described from the bilby, and it is the first identified from the Thylacomyidae family. The morphological features of the oocyst of *C. yangi* n. sp. were carefully examined and extensively compared with all identified *Choleoeimeria* sp. and *Eimeria* spp. from marsupials. As shown in Table 1, the sporulated oocysts of *C. yangi* n. sp. were morphologically different from other characterized *Choleoeimeria* sp. and *Eimera* spp. reported from marsupials (Amery‐Gale et al., 2018; Austen et al., 2014; Barker et al., 1988a, 1988b; Hillman et al., 2016; Hulst et al., 2016; Northover et al., 2019; O'Callaghan & O'Donoghue, 2001; Power et al., 2009; Yang et al., 2012). As shown in Table 1, the oocyst dimensions of *C. yangi* n. sp. (17.2 × 11.3 μm) are within a similar size range to those of *E. quokka* (18.0 × 10.8 μm), which were identified from a quokka (*Setonix brachyurus*) (Austen et al., 2014; Barker et al., 1988b) (Table 1). The oocyst of *C. yangi* n. sp. is an irregular blunt ellipsoidal shape with a L/W ratio of 1.5 whilst the oocyst of *E. quokka* is a blunt ellipsoidal shape with a L/W ratio of 1.67.

**TABLE 1:** Morphological comparison of *E. yangi* n. sp. with other Eimeria spp. in marsupials.

Coccidia	Hosts	References	Oocysts						Sporocysts				
			Shape	Measurements (μm)	Shape index	Wall (μm)	Polar granule	Oocyst residuum	Shape	Measurements	Stieda body	Substieda body	Residuum
*E. aepyprymni*	*Aepyprymnus rufescens*	Barker et al. (1988a)	Ellipsoidal	36.7 × 21.9 (32.0–42.8 × 18.4–25.2)	1.68	Bi‐layered c. 2.0	−	−	E	15.8 × 9.5 (13.6–17.6 × 7.8–10.8)	Knob‐like	Lenticulate	Compact
*E. burdi*	*Bettongia lesueur*	Hulst et al. (2016)	Spheroidal	22.6 × 14.9 (21.0–24.0 × 14.0–16.0)	1.52	Bi‐layered c. 1.0	−	−	E	11.8 × 7.4 (10.0–13.5 × 7.0–8.5)	Indistinct, domelike	Indistinct	Scattered granules
*E. angustus*	*Isoodon obesulus*	Hillman et al. (2016)	Spherical to subspherical	16.1 × 15.6 (13.1–18.5 × 13.0–18.4)	1.03	Bi‐layered c. 1.02	−	−	O	8.1 × 6.0 (6.2–9.7 × 4.7–7.6)	Absent	Absent	Compact
*E. gaimardi*	*Bettongia gaimardi*	Barker et al. (1988a)	Ovid to pyriform	34.6 × 24.3 (32.0–39.2 × 20.8–26.4)	1.4	Bi‐layered c. 1.6	−	−	O	15.0 × 9.6 (13.6–16.0 × 9.0–10.4)	Small	Lenticulate	Loose aggregate of granules
*E. hypsiprymnodontis*	*Hypsiplymnodon moschatus*	Barker et al. (1988a)	Bluntly ellipsoidal	28.6 × 22.7 (225.6–32.0 × 20.8–24.0)	1.26	Bi‐layered c. 3.0	+	−	E	17.0 × 8.3 (16.0–17.6 × 8.0–8.8)	Knob‐like	Prominent	Irregular‐shaped
*E. kairiensis*	*Hypsiplymnodon moschatus*	Barker et al. (1988a)	Spherical to subspherical	13.4 × 12.9 (11.2–15.2 × 10.0–15.2)	1.04	Bi‐layered c. 0.6	+	−	E	8.2 × 5.2 (6.4–9.6 × 4.0–6.4)	Knob‐like	Absent	Scattered granules
*E. mundayi*	*Potorous tridactylus*	Barker et al. (1988a)	Spherical to subspherical	16.9 × 16.2 (13.6–20.8 × 13.6–19.2)	1.04	Bi‐layered c. 1.4	+	−	E	9.7 × 6.2 (8.0–12.0 × 4.8–8.0)	Small	Small	Scattered granules
*E. potoroi*	*Potorous tridactylus*	Barker et al. (1988a)	Ovid to pyriform	26.2 × 18.5 (24.0–29.6 × 16.8–22.4)	1.4	Bi‐layered c. 1.4	−	+	E	11.9 × 7.6 (10.8–13.6 × 6.4–8.8)	Protuberant	Lenticulate	Scattered granules
*E. quokka*	*Setonix brachyurus*	Barker et al. (1988b), Austen et al. (2014)	Bluntly ellipsoidal	18.0 × 10.8 (13.6–21.6 × 8.8–15.2)	1.67	Bi‐layered c. 1.4	+	−	E	7.7 × 5.0 (6.4–9.6 × 4.0–6.4)	Small	Absent	Tight granular
*E. setonicis*	*Setonix brachyurus*	Barker et al. (1988b), Austen et al. (2014)	Ellipsoidal to cylindroid	29.9 × 17.9 (26.4–33.6 × 16.8–19.2)	1.67	Bi‐layered c. 1.4	+	−	E	12.0 × 7.7 (10.4–13.6 × 7.2–8.8)	Protuberant	Lenticulate	Tight granular
*E. spearei*	*Hypsiplymnodon moschatus*	Barker et al. (1988a)	Spherical to subspherical	16.9 × 16.3 (15.2–20.0 × 13.6–18.4)	1.04	Bi‐layered c. 1.2	+	−	E	10.4 × 6.4 (8.0–12.0 × 5.2–7.2)	Knob‐like	Small	Aggregate of granules
*E. spratti*	*Hypsiplymnodon moschatus*	Barker et al. (1988a)	Subspheroidal	21.3 × 13.1 (18.4–24.0 × 16.0–23.2)	1.63	Bi‐layered c. 2.0	+	−	E	13.1 × 7.6 (11.2–15.2 × 6.0–8.8)	Inconspicuous	Clear	Compact
*C. taggarti*	*Antechinus flavipes*	Amery‐Gale et al. (2018)	Spheroidal	15.6 (14.3–16.9)	1	One‐layered	−	−	E	9.0 × 7.0 (7.5–10.1 × 6.2–7.8)	Absent	Absent	Prominent
*E. tinarooensis*	*Hypsiplymnodon moschatus*	Barker et al. (1988a)	Subspherical to bluntly ellipsoidal	26.0 × 23.8 (24.0–28.0 × 21.6–26.4)	1.09	Bi‐layered c. 3.6	+	−	E	13.3 × 7.9 (11.2–14.4 × 7.2–8.4)	Knob‐like	Clear	Loose aggregate of granules
*E. trichosuri*	*Trichosurus cunninghami*	O'Callaghan and O'Donoghue (2001)	Ellipsoidal to cylindrical	41.4 × 22.7 (34.4–49.2 × 18.4–27.8)	1.8	One‐layered c. n.a.	+	+	E	15.6 × 9.9 (13.9–18.0 × 8.2–12.0)	Yes, n.a	Yes, n.a.	Yes, n.a.
*E. trichosuri*	*Trichosurus cunninghami*	Power et al. (2009)	Ovid	40.3 × 20.8 (31.5–45.9 × 17.7–23.6)	1.9	Bi‐layered c. n.a.	+	+	E	13.9 × 9.6 (9.6–18.8 × 7.3–12.0)	Yes, n.a	Yes, n.a.	Yes, n.a.
*E. wilcanniensis*	Macropus rufus	Yang et al. (2012)	Ovid	31.8 × 20.3 (28.0–37.8 × 18.4–23.6)	1.57	Bi‐layered c. 1.0	−	−	O	11.9 × 9.3 (9.5–14.3 × 8.2–10.9)	Absent	Absent	
*E. woyliei*	*Bettongia penicillata*	Northover et al. (2019)	Pyriform	36.7 × 26.3 (31.6–40.8 × 22.6–31.0)	1.4	Bi‐layered c.1.6	+	−	E	15.6 × 10.3 (13.3–17.8 × 9.0–12.3)	Indistinct dome‐like	Indistinct dome‐like	Irregular‐shaped
*C. yangi* n. sp.	*Macrotis lagotis*	This study	Irregular blunt ellipsoidal	17.2 × 11.3 (17.0–18.0 × 11.0–12.0)	1.5	Bi‐layered c. 0.9	+	−	O	7.8 × 5.7 (7.0–8.0 × 5.0–6.0)	Absent	Absent	Scattered granules

*Note*: −: absent; +: present.

Abbreviations: E, ellipsoidal; O, ovoidal.

Molecular characterization of *C. yangi* n. sp. at the 18S rRNA and COI loci revealed that it did not match any of the sequences of *Choleoeimeria* spp. and *Eimeria* spp. in the GenBank. The 18S rRNA sequence of *C. yangi* n. sp. shared the most genetic similarity with *E. angustus* (KU248093) and *E. taggarti* (MK809543) while at the COI locus it was unique, with only a 90.6% genetic similarity to *C. taggarti* (MK813349).

Currently, the application of molecular tools in coccidian taxonomy is playing an important role as can be seen by the examples of *E. taggarti* and *C. taggarti* mentioned in this paper. Amery‐Gale et al. (2018) first characterized this coccidia as *E. taggarti*. Kruth et al. (2020) then refined the taxonomic placement of *E. taggarti* as *C. taggarti* based on the full mitochondrial genome and nuclear 18S rDNA sequence alignment. *E. taggarti* was the first *Eimeria* sp. renamed as a *Choleoeimeria* sp. from marsupials. The characterized *C. yangi* n. sp. from the bilby in this study further confirms that *Choleoeimeria* spp. are not limited to reptiles. The high oocyst morphological similarity between *Eimeria* spp. and *Choleoeimeria* spp. suggests that some of the *Eimeria* spp. identified from marsupials might be *Choleoeimeria* spp. The rapid development of sequencing techniques will result in more molecular data becoming available in future, and this will make coccidian molecular taxonomy more reliable and achievable. Meanwhile, the traditional taxonomy skills based on oocyst morphological characters provide valuable information and should still be performed. For example, Matsubara et al. (2017) molecularly characterized *Isospora* spp. in the domestic pigeon (*Columba livia domestica*, Gmelin, 1789) in Japan. The authors did not conduct a morphological study and only carried out PCR, sequencing, and cloning to get the pure sequences. The 18S rRNA sequences (5) they submitted to the GenBank were all called *Isospora* spp. However, only two grouped in the *Isospora* clade and the other three grouped in the *Eimeria* clade (Yang, Brice, Elloit, & Ryan, 2016). Similar examples will undoubtedly be picked up from other journal publications.

## 5 CONCLUSION

Morphological and genetic comparison of *C. yangi* with other known *Choleoeimeria* spp. and *Eimeria* spp. revealed that the coccidian identified in the captive‐bred bilby is a new species. *C. yangi* is the first coccidian to be reported from a bilby in Australia and is the first sequence available of a coccidian from this marsupial species. The morphological and genetic sequence information from our study of *C. yangi* further contributes towards our knowledge of those *Eimeria* spp. infecting the order Peramelemorphia.

## AUTHOR CONTRIBUTIONS

**Belinda Brice:** Conceptualization (equal); data curation (equal); formal analysis (equal); investigation (equal); validation (equal); writing – original draft (equal); writing – review and editing (equal). **Huimin Gao:** Data curation (equal); investigation (equal); validation (equal). **Bruno P. Berto:** Conceptualization (equal); investigation (equal); methodology (equal); validation (equal); writing – review and editing (equal). **Gwyneth Thomas:** Investigation (equal); methodology (equal); project administration (equal). **Aileen Elloit:** Formal analysis (equal); investigation (equal); methodology (equal). **Alireza Zahedi:** Conceptualization (equal); investigation (equal); methodology (equal); supervision (equal); writing – review and editing (equal).

## CONFLICT OF INTEREST STATEMENT

The authors have no conflicts of interest to declare. All co‐authors have seen and agree with the contents of the manuscript, and there is no financial interest to report.

## Data Availability

The 18S rRNA and COI additional sequence data generated from this study are accessible in the public domain: https://www.ncbi.nlm.nih.gov/nuccore under the GenBank accession numbers of OR543995 and ON894305 for the 18S rRNA and COI loci, respectively, after the paper is published. The 18S and COI additional sequence data used in the phylogenetic analysis were derived from the following resources available in the public domain: https://www.ncbi.nlm.nih.gov/nuccore with the GenBank accession numbers in Figures 3a,b and 4a,b.
